# Rapid and substantial increases in anticoagulant use and expenditure in Australia following the introduction of new types of oral anticoagulants

**DOI:** 10.1371/journal.pone.0208824

**Published:** 2018-12-06

**Authors:** Alice Morgan, Grace Joshy, Andrea Schaffer, Tracey-Lea Laba, Melisa Litchfield, Sallie Pearson, Emily Banks

**Affiliations:** 1 National Centre for Epidemiology and Population Health, Australian National University, Canberra, Australia; 2 Centre for Big Data Research in Health, University of New South Wales, Sydney, Australia; 3 Menzies Centre for Health Policy, The University of Sydney, Sydney, Australia; Monash University, AUSTRALIA

## Abstract

**Objectives:**

To quantify changes in anticoagulant use in Australia since the introduction of Non-vitamin K antagonist anticoagulants (NOACs) and to estimate government expenditure.

**Design:**

Interrupted-time-series analysis quantifying anticoagulant dispensing, before and after first Pharmaceutical Benefits Scheme (PBS) NOAC listing in August 2009 for venous thromboembolism prevention; and expanded listing for stroke prevention in non-valvular atrial fibrillation (AF) in August 2013, up to June 2016. Estimated government expenditure on PBS-listed anticoagulants.

**Setting and participants:**

PBS dispensing in 10% random sample of Australians, restricted to continuous concessional beneficiaries dispensed oral anticoagulants from July 2005 to June 2016. Total PBS anticoagulant expenditure was calculated using Medicare Australia statistics.

**Main outcome measures:**

Monthly dispensing and initiation of oral anticoagulants (warfarin, rivaroxaban, dabigatran or apixaban). Annual PBS anticoagulant expenditure.

**Results:**

An estimated 149,180 concessional beneficiaries were dispensed anticoagulants (100% warfarin) during July 2005. This increased to 292,550 during June 2016, of whom 47.0%, 27.1%, 18.7% and 7.2% were dispensed warfarin, rivaroxaban, apixaban and dabigatran, respectively. Of 16,500 initiated on anticoagulants in June 2016, 24.3%, 38.2%, 30.0% and 7.5% were initiated on warfarin, rivaroxaban, apixaban, and dabigatran, respectively. Compared to July 2005-July 2013, from August 2013-June 2016, dispensings for all anticoagulants increased by 2,303 dispensings/month (p<0.001, 95%CI = [1,229 3,376]); warfarin dispensing decreased by 1,803 dispensings/month (p<0.001, 95%CI = [–2,606, –1,000]). Total PBS anticoagulant expenditure was $19.5 million (97.0% concessional) in 2008/09, of which 100% was warfarin and $203.3 million (86.2% concessional) in 2015/16, of which 11.2% was warfarin.

**Conclusions:**

The introduction of the NOACs led to substantial increases in anticoagulant use and expenditure in Australia.

## Introduction

Thrombotic disorders, including stroke and venous thromboembolism (VTE) contribute to significant morbidity and mortality globally. Stroke is the second most common cause of death, contributing to 10.1% of all deaths globally in 2016, and a major cause of disability worldwide [1]. Atrial fibrillation (AF) is the most important cause of cardioembolic stroke, the most severe subtype of ischaemic stroke, and is associated with an up to 5-fold increased risk of stroke [2]. VTE, of which approximately one-third presents as pulmonary embolism and the remainder as deep vein thrombosis, contributes to approximately 10% of all hospital deaths, making it the single largest preventable cause of death in hospitalised patients [3]. Given the significant mortality and morbidity burden of stroke and VTE, effective prophylaxis with oral anticoagulants (OACs), including warfarin, and the non-vitamin k oral anticoagulants (NOACs) is essential.

Warfarin is the most extensively used OAC in the world, with 1-2% of adults in the developed world estimated to have been prescribed warfarin [4–7]. When used optimally, it is highly efficacious, providing a relative risk reduction for stroke in patients with AF of 64% compared to placebo [8]. However, warfarin requires complex management that is often complicated because of its multiple interactions with foods and other medicines [9, 10]. Furthermore, there are significant risks with taking warfarin: up to 2% of people treated will experience a major bleed, and between 0.1 and 0.5% will have an intracranial bleed [11–13].

There is extensive under-treatment of those at risk of stroke; at least one-third of patients with AF and other known risk factors for stroke who are candidates for warfarin therapy do not receive it [14]. NOACs–including rivaroxaban, dabigatran and apixaban–were listed on the Australian Pharmaceutical Benefits Scheme (PBS) for prevention of VTE from August 2009 and for stroke prevention in AF from August 2013 to address this unmet need in anticoagulant therapy. NOACs have many theoretical advantages over warfarin including comparable or superior efficacy in trial populations (e.g. in AF, reduced risk of stroke, thromboembolism, and all-cause mortality) and reduced monitoring requirements [15, 16]. However, NOACs are also considerably more costly than warfarin [17].

Despite their importance, evidence on the use of NOACs and their consideration as an alternative to warfarin in the treatment of thrombotic conditions in Australia is limited. The only previous relevant study, to our knowledge, examined the uptake of NOACs in the Australian Veterans’ population. It found rapidly increasing use of NOACs in this population following their PBS listing for stroke prevention in AF and concluded that this may reflect use in those previously contraindicated to warfarin [18]. However, the veterans’ population is very elderly and has patterns of health services use that differ from the broader population, so evidence on use in a more mainstream population is required.

The aim of this study is to quantify changes in OAC use in Australia using a representative 10% sample dataset of the PBS and to estimate total PBS OAC expenditure.

## Methods

### Context and setting

The PBS is Australia’s national medicine subsidy program, subsidising approximately 75% of prescribed medicine use in Australia [19]. The PBS 10% sample used in this analysis is a standardised, longitudinal, unit-record extract containing all PBS medicine dispensing data for a random 10% sample of Australians (approximately 2.5 million people) that is made available for research purposes by the Department of Human Services [19].

**Fig 1:**
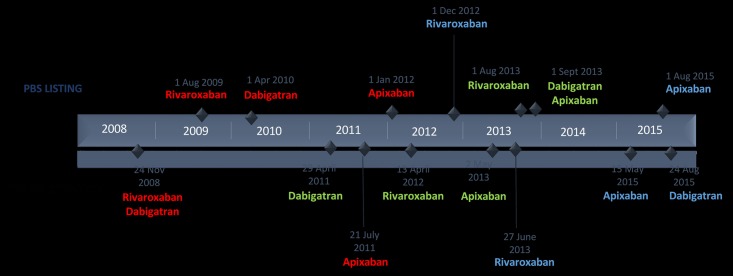
Timeline of NOAC market entry in Australia. Notes: The dates shown above the timeline are the PBS listing (reimbursement) dates while the dates shown below the timeline are the Therapeutic Goods Administration (TGA) registration (market approval) dates for each of the approved indications for NOAC use. Legend: Red: prevention of venous thromboembolic events (VTE) in adult patients who have undergone elective total hip or total knee replacement surgery; Green: prevention of stroke and systemic embolism in (adult) patients with non-valvular atrial fibrillation (AF) and at least one additional risk factor for stroke; Blue: Treatment (Tx) of deep vein thrombosis (DVT) and pulmonary embolism (PE) and for the prevention (Pv) of recurrent DVT and PE in adults.

All PBS medicines are assigned Anatomical Therapeutic Chemical (ATC) codes, as well as unique PBS item codes that provide medicine details at the product level, including approved indication, where applicable [19]. PBS item codes for NOACs (but not warfarin) provided a proxy of the indication for use. Fig 1 provides an overview of the introduction of the NOACs onto the Australian market for each of their approved indications for use.

### Study population

To maintain consistency, the study population included continuous concessional beneficiaries (individuals dispensed medicines attracting a concessional co-payment during the entire study) dispensed anticoagulants. The PBS database does not capture data on medicines priced lower than the patient co-payment until at least April 2012, and thereby under-ascertains the utilisation of certain medicines prior to this time [19]. As the concessional co-payment threshold is lower than the cost of nearly all medicines on the PBS, restricting to the concessional population allows for more complete capture of medicine use over time.

At the beginning of the study period (during July 2005), 51.7% of the (14,918) dynamic 10% sample cohort dispensed OACs were male, 83.3% were aged 65 years and older, and 100% were dispensed warfarin. At the end of the study period (during June 2016), 54.9% of the (29,255) cohort were male, 90.2% were aged 65 years and older, and 46.0% were dispensed warfarin. The dynamic nature of the study population did not allow for age-adjustment at a population level.

### Study variables

This analysis included all OAC (warfarin, rivaroxaban, dabigatran and apixaban) dispensing records (for continuous concessional beneficiaries) during the study period of 1 July 2005 to 30 June 2016. OACs were identified by ATC code: warfarin (B01AA03); rivaroxaban (B01AF01); dabigatran (B01AE07); and apixaban (B01AF02).

### Statistical analysis

We calculated the number and proportion of Australian concessional beneficiaries dispensed each OAC for each month of the study period using the PBS 10% sample and extrapolating the findings to the overall PBS (continuous) long-term concessional population by multiplying the population by ten-fold.

**Fig 2:**
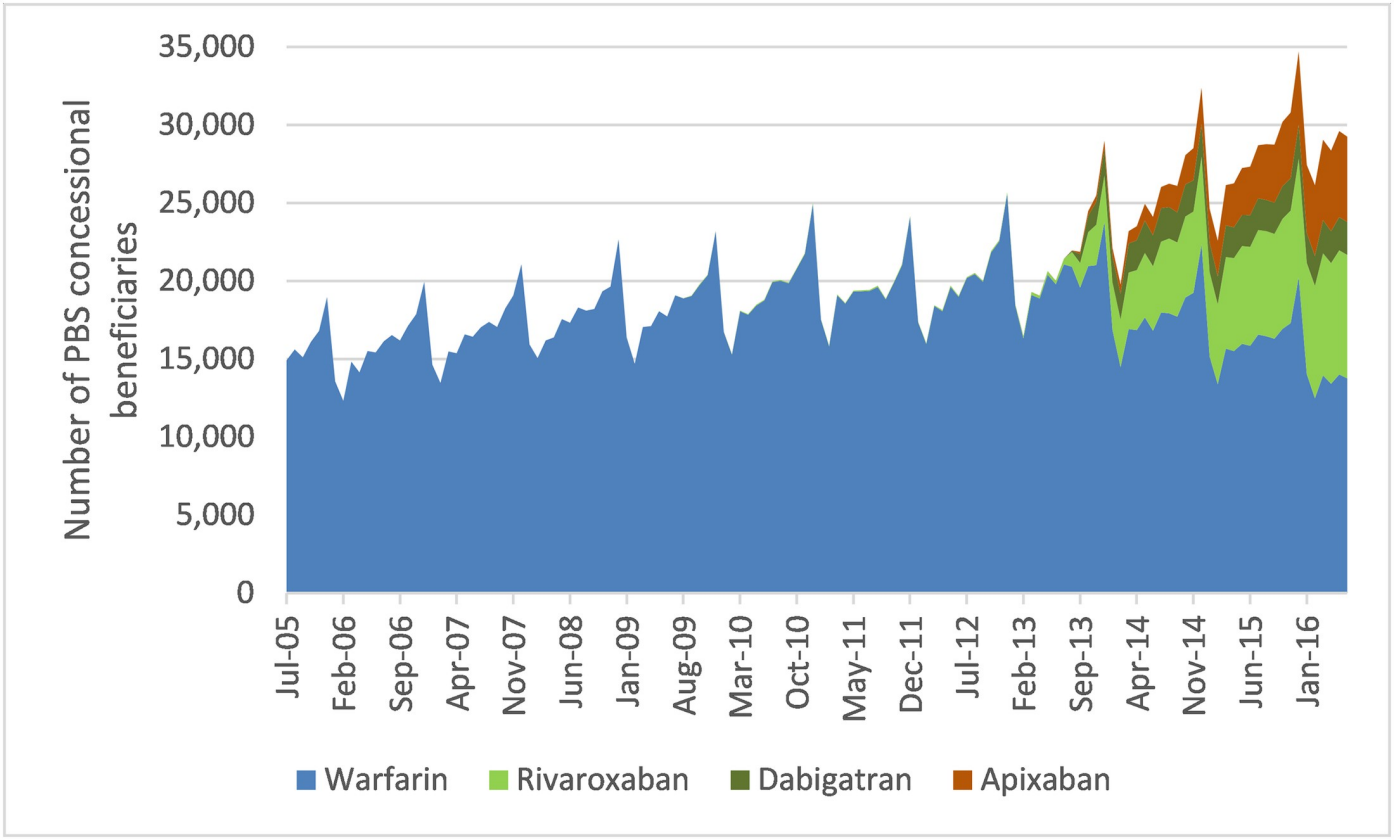
Number of PBS concessional beneficiaries’ dispensed anticoagulants by month (July 2005–June 2016).

The number and proportion of OAC users initiating a new course of each of the OACs (‘initiators’), defined as individuals with no prior dispensings during the study period for at least one year, including individuals with more than one year between dispensings of the same or another anticoagulant, was also quantified. To compare changes in warfarin and total anticoagulant (warfarin and NOAC) dispensing following the introduction of the NOACs, an interrupted time series analysis (ITSA) was performed using the Stata ‘ITSA’ command, described elsewhere [20]. Two intervention periods were examined: the initial listing of the first NOAC (rivaroxaban) in August 2009; and following the expanded indication for stroke prevention in AF in August 2013 (for rivaroxaban; September 2013 for dabigatran and apixaban) (Fig 2). The first intervention period (August 2009 to July 2013) was compared with the pre-intervention period (July 2005 to July 2009), and the second intervention period (August 2013 to June 2016) was compared with the first intervention period (August 2009 to July 2013). The pre-intervention trend (slope) projected into the subsequent treatment period serves as the counterfactual and is statistically compared to provide an estimate of the effect of the interruption. Post-trend analysis estimates the post-intervention trends separately after the first and second intervention periods.

Autocorrelation and seasonality between time points were tested and found to be present. Seasonality was adjusted for using the Holt-Winters seasonal smoothing approach and the Prais-Winsten ordinary least-squares regression approach (‘prais’ command in Stata) was used to adjust for autocorrelation. Stata version 14.1 was used to perform all analyses.

### PBS expenditure

Total PBS expenditure on anticoagulants was separately calculated using Medicare Australia PBS item reports, which produce expenditure statistics based on requested PBS item codes and by patient category (general or concessional) [21]. PBS expenditure is reported as expenditure in the relevant year.

## Results

### Use

An estimated 149,180 concessional beneficiaries were dispensed OACs during July 2005, the beginning of the study period, of whom 100% were dispensed warfarin. This increased to 292,550 in June 2016, of whom 47% were dispensed warfarin (Fig 2).

The proportion dispensed warfarin comprised more than 98% of concessional beneficiaries dispensed OACs prior to August 2013. However, this decreased substantially in the first year of the expanded NOAC listing from 95.2% (209,150/219,680) during August 2013 to 69.1% (179,860/260,320) during July 2014.

Rivaroxaban dispensing increased from 1.7% (3,650/214,340) of those dispensed OACs during July 2013 to 4.3% (9,540/219,680) during August 2013, and to 27.1% (79,130/292,550) in June 2016. The proportion dispensed dabigatran was higher than those dispensed apixaban until October 2014, when this observation was reversed. The proportion dispensed apixaban continued to increase for the remainder of the study period to 18.7% (54,680/292,550), while those dispensed dabigatran plateaued and were at 7.2% (21,120/292,550) in June 2016.

Rivaroxaban remained the most commonly dispensed NOAC throughout the study period while warfarin remained the most commonly dispensed OAC overall, although use is declining and the number of people dispensed NOACs exceeds warfarin. Dispensings for NOACs were predominantly for use in stroke prevention in AF. From September 2013, <5% of monthly NOAC dispensing was for prevention of VTE in patients undergoing hip or knee replacement.

### Initiation

**Fig 3:**
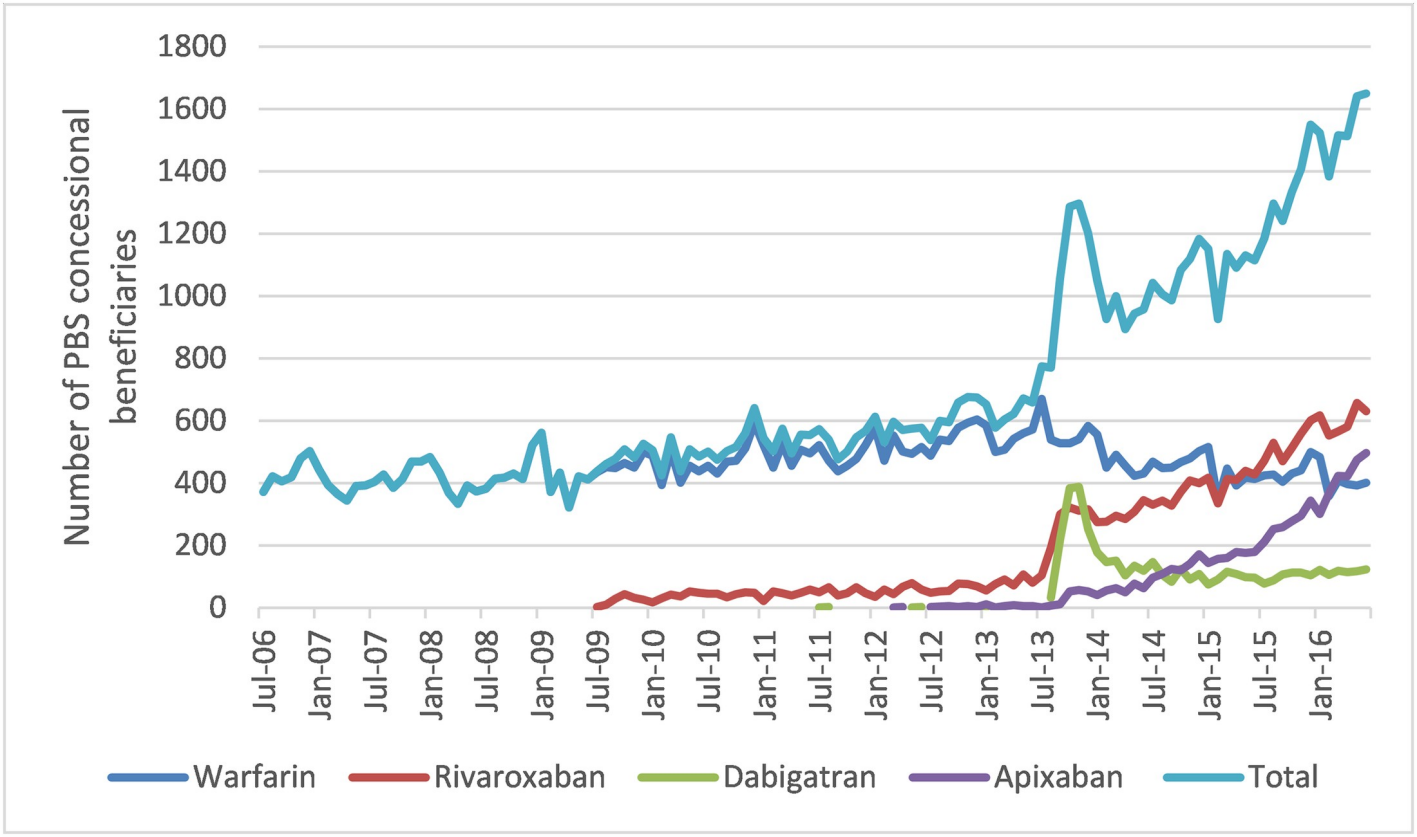
Number of people initiating anticoagulants by month.

The number of people initiating OACs per month during July 2006 was 3,710, all of whom were initiated on warfarin. In June 2016, 16,500 people were initiated on OACs of whom 24.3% were initiated on warfarin (Fig 3).

The proportion of people initiating on warfarin decreased from 86.5% (6,700/7,750) in July 2013 to <50% (5,280/10,570) in September 2013, coinciding with the expanded PBS listing of NOACs for stroke prevention in AF (August 2013).

At the end of the study period, rivaroxaban was the most commonly initiated OAC with 6,300 (38.2%) of the 16,500 people initiating OACs during the month of July 2016, followed by 4,960 (30.0%) with apixaban, 4,010 (24.3%) with warfarin and 1,230 (7.5%) with dabigatran.

### Changes in OAC use

**Fig 4:**
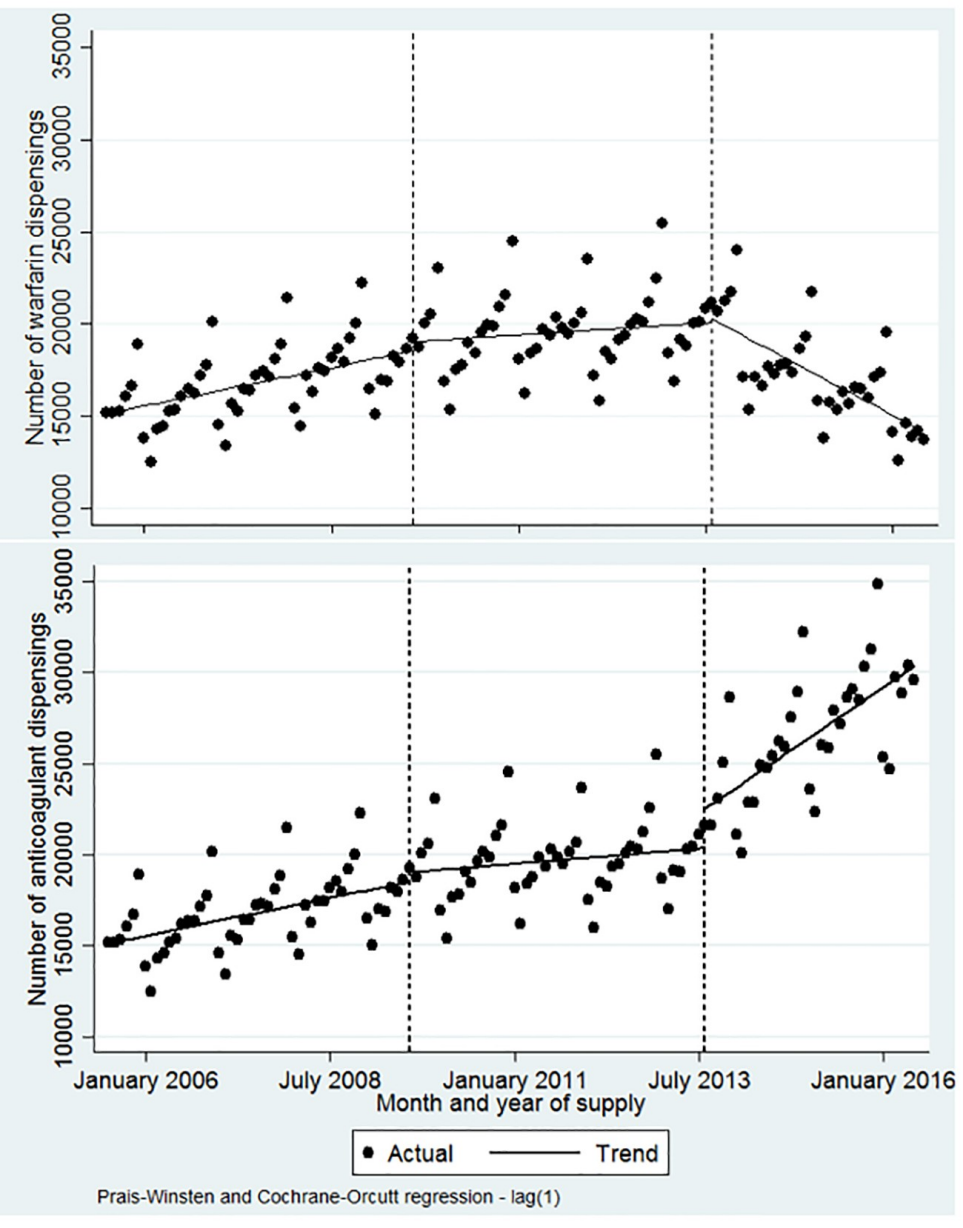
Interrupted time series analysis of warfarin and all anticoagulant dispensings.

ITSA showed that from July 2005 to August 2009, the level and trend of OAC dispensing was approximately 151,510 per month, increasing significantly by 692 dispensings per month (p<0.001, 95% CI = [282, 1,103]) over this period (Fig 4). Following the initial introduction of the NOACs on the PBS for VTE prophylaxis following major orthopaedic surgery (August 2009), no significant change in the level or trend of anticoagulant (or warfarin) dispensing was seen. While this was not unexpected given that warfarin is available for a broader range of indications (such as coronary occlusion), it does provide a useful comparison of the more limited use of NOACs in VTE prophylaxis compared with its expanded indication over time. Following the second intervention period, which represents the expanded indication in stroke prevention in AF (August 2013), dispensing of warfarin decreased significantly at a rate of 1,803 dispensings per month (p<0.001, 95% CI = [–2,606, –1,000]), while dispensing of OACs (warfarin and NOACs) increased significantly at a rate of 2,303 dispensings per month (p<0.001, 95% CI = [1,229, 3,376]).

### PBS expenditure

While the costs of PBS medicines can change over time depending on PBS policies, NOACs are considerably more costly to the PBS than warfarin. At the end of the study period (June 2016), the dispensed price for warfarin was $15.38–$16.71 per pack, depending on the dosage dispensed (1 mg–5mg, respectively). While the dispensed price for rivaroxaban was $37.59 –$124.54, dabigatran was $36.19 –$109.12, and apixaban was $39.14 –$96.58, per pack depending on the dosage and quantity dispensed.

**Fig 5:**
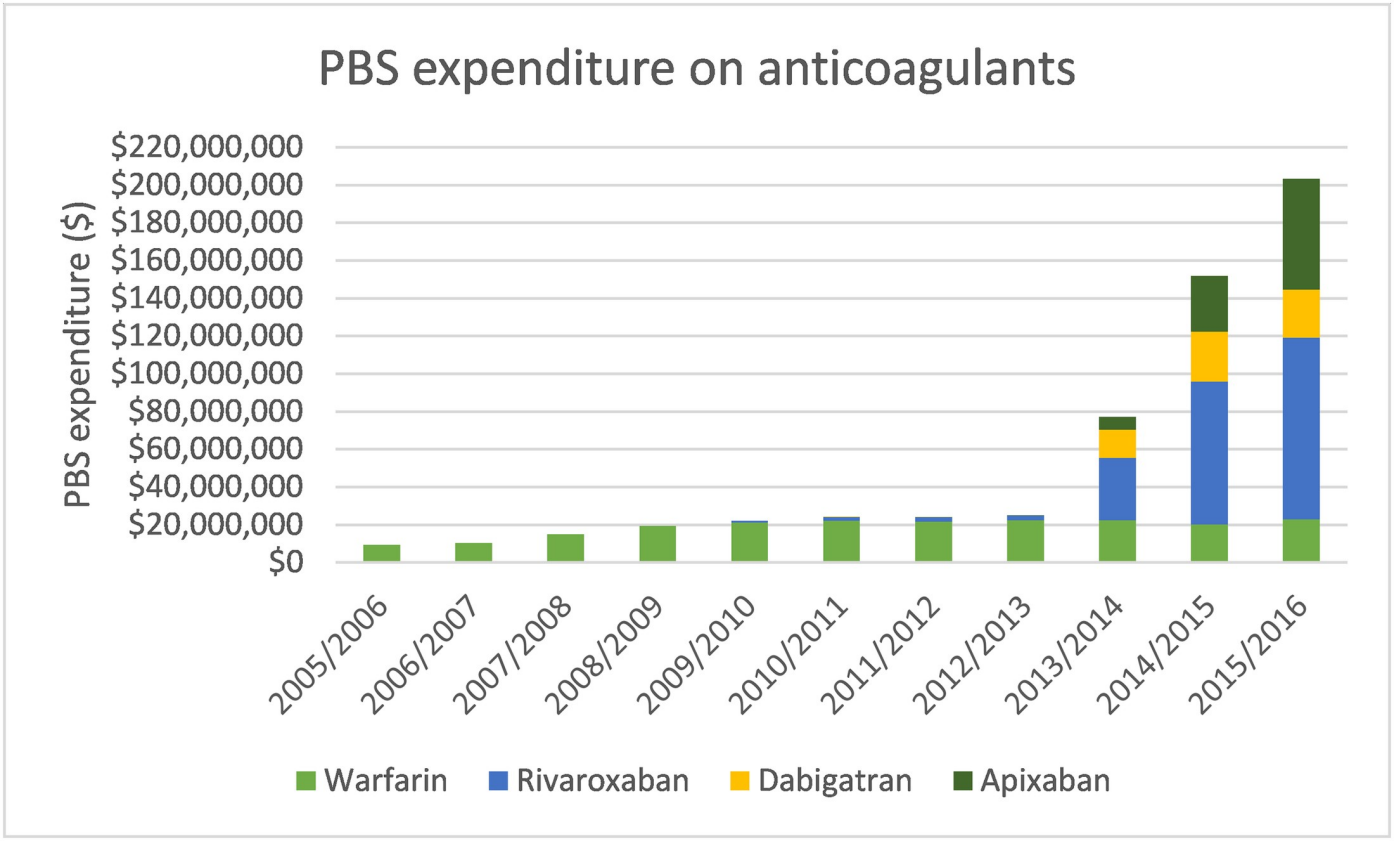
PBS expenditure on anticoagulants (2005/06–2015/16).

Annual PBS expenditure on OACs increased from $9.3 million (97.3% concessional) in 2005/06 to 19.5 million (97.0%) in 2008/09, of which 100% was on warfarin, to $203.3 million (86.2% concessional) in 2015/16, of which 11.2% was warfarin, 47.4% rivaroxaban, 12.5% dabigatran and 28.9% apixaban (Fig 5). PBS expenditure increased more than 8-fold from $25.1 million (94.3% concessional) in the year prior to the expanded NOAC indication (2012/13) compared to $203.3 million (86.2% concessional) in 2015/16.

## Discussion

The expanded listing of the NOACs onto the Australian PBS for stroke prevention in AF had a rapid and substantial impact on OAC dispensing. We found that the gradually increasing use of OACs more than tripled following the expanded NOAC listing, and increases in monthly OAC dispensing far exceeded corresponding decreases in monthly dispensing of warfarin by approximately 28%. Furthermore, three quarters of new use was in patients initiating on NOAC, with the majority of patients initiating on rivaroxaban or apixaban at the end of the study period. Taken together, this indicates that, a substantial proportion of the increase was attributed to additional patients being dispensed anticoagulants as opposed to switching from warfarin to a NOAC. This finding likely reflects use in a subset of the population previously unable to take warfarin, in addition to new patients being preferentially commenced on NOACs.

Our findings of OAC use in a broader Australian population using a representative 10% sample dataset corroborate international data demonstrating rapid adoption of NOACs in clinical practice and rapid increases in anticoagulant expenditure and are consistent with a previous Australian study in the Veterans’ population [22–24, 18].

PBS expenditure on anticoagulants increased more than 8-fold following the expanded PBS listing of the NOACs from $25.1 million in 2012/13 to $203.3 million in 2015/16, consistent with the changing pattern of NOACs observed in our analysis. However, the potential cost savings generated from reductions in health service use including laboratory monitoring and MBS consultations that are likely associated with NOACs but not warfarin, are not reflected in our expenditure estimates.

Nevertheless, it is important to consider whether the increased use of NOACs and associated expenditure that we have observed in practice is delivering value for money. While we have not measured health benefits, we note that a recent Australian Government *Review of Anticoagulation Therapies in Atrial Fibrillation*, recommended the restriction of NOACS to use in people unable to tolerate warfarin to mitigate uncertainties regarding the magnitude of any incremental clinical and cost-effectiveness benefit of NOACs over other therapies, and the total high unpredicted cost [25]. Neither Australian clinical guidelines, PBS listings nor, as shown by our findings, clinical practice presently adopted these recommendations. However, this is important to consider in the context of the potential for savings in terms of reductions in health service usage from preventing strokes given their significant burden.

The main strengths of this study are its large, representative sample and detailed independent administrative information on medicine dispensing in the Australian population. We used an ITSA design to assess statistically the impact that the NOACs had on the level and trend of anticoagulant dispensing in the PBS concessional population using the PBS 10% sample.

To maintain consistency in the population over time, the study was restricted to concessional beneficiaries only and excluded general beneficiaries and anyone with a change in concessional status over the study period thereby potentially substantially underestimating dispensing trends. However, as most people aged 65 years and older are concessional, they also represent the primary users of OACs. While the PBS 10% sample provides useful information about dispensing in the Australian population, it does not provide clinical information on medicine use, therefore no conclusions can be made about the intended (e.g. fewer strokes) and unintended (e.g. increased bleeds) outcomes of anticoagulant use.

The expanded PBS listing of the NOACs for stroke prevention in AF rapidly accelerated steadily increasing use of PBS anticoagulant dispensing and expenditure. At least some of these changes may reflect use in Australians previously unwilling or unable to take warfarin, increasing the overall anticoagulant-treated population. However, this needs to be considered in the context of the substantial economic impact that the listing of the NOACs has had on PBS anticoagulant expenditure. Post-market experience with NOACs, including analyses of administrative data, has the potential to help inform ongoing cost-effective use of NOACs. With further market experience and post-market safety and efficacy surveillance, we may be able to evaluate whether this investment has contributed to lessening the burden of stroke and VTE in Australia.

### Ethics and data access approvals

The study has ethics approval from the New South Wales Population and Health Services Research Ethics Committee (2013/11/494). All data were de-identified, and thus the requirement for informed consent was waived with approval from the NSW Population and Health Services Research Ethics Committee. The Australian Department of Human Services External Request Evaluation Committee approved access to the data.

This research was conducted using Pharmaceutical Benefits Scheme (PBS) data collected by the Australian Government Department of Human Services (DHS). Researchers may apply for restricted access to these data through a licence agreement with the appropriate data custodian and ethical approvals.
